# Acupotomy therapy for cervical vertigo: A protocol for a systematic review and meta-analysis

**DOI:** 10.1097/MD.0000000000020662

**Published:** 2020-07-17

**Authors:** Rongfang Xie, Jianyu You, Liting Liu, Chunhua Huang, Yu Liang

**Affiliations:** a Jiangxi University of Traditional Chinese Medicine; b Affiliated Hospital of Jiangxi University of Traditional Chinese Medicine, Nanchang, China.

**Keywords:** acupotomy, cervical vertigo, protocol, systematic review

## Abstract

**Background:**

Acupotomy has been widely used to relieve cervical vertigo (CV). However, the efficacy of acupotomy for CV is uncertain. The purpose of this study is to evaluate the efficacy and safety of the acupotomy for CV.

**Methods:**

The following electronic databases will be searched to identify relevant randomized controlled trials (RCTs) for inclusion in the review from inception to April 2020: PubMed, the Cochrane Library, Embase, the China National Knowledge Infrastructure, Wanfang Database, Chinese Science and Technology Periodical Database, and Chinese Biomedical Literature Database. Two researchers will independently select studies, collect data, and assess the methodology quality by the Cochrane risk of bias tool. Meta-analysis will be completed by RevMan V.5.3 software.

**Results:**

This systematic review will provide an assessment of the current state of acupotomy for CV, aiming to assess the efficacy and safety of acupotomy for Patients with CV.

**Conclusion:**

This systematic review will provide a credible Evidence-based for the clinical treatment of CV with acupotomy.

PROSPERO registration number: CRD42019134712.

## 1 Introduction

Vertigo is one of the 20 most common causes of medical consultation for adult patients. In 80% of cases, the symptoms are very serious and require medical intervention.^[1]^ Among them, cervical vertigo (CV) is a form of vertigo caused by neck disease.^[2,3]^ The main clinical symptoms of CV are dizziness, blurred vision, neck pain, nausea, vomiting, and fainting when turning the head or bending the neck laterally to a specific position.^[4]^ Studies have shown that CV may be related to degenerative changes in the cervical spine, and its pathological basis is vertebral artery stenosis and insufficient blood supply, so in the elderly, cervical spondylosis with vertigo is more common.^[5]^ According to statistics, CV affects about 30% of the elderly in the world.^[6]^ At the same time, as peoples lifestyles change, the incidence of CV is rising and tending to be younger. CV is prone to repeated attacks and can develop into a severe stroke, which brings greater challenges to clinical treatment,^[7]^ and CV can also lead to increased healthcare costs and decreased patient quality of life and work performance.^[8]^

Regarding the treatment management of CV, the current first-line treatment is mainly including drug and surgery therapy. These treatment methods have been used for the management of CV, but it is difficult to obtain satisfactory results, and the cost is high, usually accompanied by certain adverse reactions. Therefore, there is an urgent need to find a safer and more effective alternative therapy.^[9,10]^

Acupotomy, also known as a mini-scalpel needle or needle-knife, is a new type of therapy that combines Chinese meridian theory with modern surgical techniques. This therapy was invented by Professor Zhu Hanzhang.^[11]^ It absorbs modern pathology and anatomy theory as well as anesthesia and aseptic techniques.^[12]^ Acupotomy can effectively eliminate the adhesion and contracture, reduce the tension of the soft tissue, and restore the normal function of the tissue by performing appropriate loosening operations on the diseased soft tissue, such as cutting and peeling, and has the characteristics of high safety, less trauma, less complications, and low cost.^[13,14]^ Therefore, acupotomy has been widely used by doctors in the orthopaedics and pain departments for clinical treatment, with satisfactory results.^[15]^

Although the benefit of acupotomy has been widely proven and accepted in clinical practice, the effectiveness of acupotomy treatment of CV is still controversial. This study uses the method of evidence-based medicine to analyze and evaluate the RCTs of patients with CV treated by acupotomy, and provides a basis for further improving the clinical efficacy of CV patients. The study will evaluate the effectiveness and safety of acupotomy treatment of CV patients.

## 2 Methods

### 2.1 Inclusion criteria for study selection

#### 2.1.1 Types of studies

All RCTs of acupotomy treatment for CV will be included without language restriction. Observational studies, cross-over studies, conference abstracts, animal studies, and letters will be excluded.

#### 2.1.2 Types of participants

Participants with CV must be diagnosed with a standard diagnostic criteria. There are no limits to research subjects age, gender, race, condition duration, or intensity. Participants with serious underlying diseases who are not suitable for acupotomy treatment will be excluded.

#### 2.1.3 Types of interventions

##### 2.1.3.1 Experimental interventions

Acupotomy therapy which only included acupotomy alone, regardless of different acupoints or needle materials. Mixed therapies were excluded.

##### 2.1.3.2 Control interventions

The control group will receive an internationally recognized therapy such as conventional pharmacological therapies. No treatment, placebo, and acupuncture will also be included. Studies that compare the effect of different types of acupotomy therapy will be excluded.

#### 2.1.4 Types of outcome measures.

##### 2.1.4.1 Primary outcomes

Cervical vertigo symptoms and functional rating scale (ESCV), visual analogue scale (VAS) and Percentage of Clinical Efectiveness will be accepted as the primary outcomes.

##### 2.1.4.2 Additional outcomes

Secondary outcomes will include a safety assessment, as judged by incidence rate and severity of adverse effects

### 2.2 Search methods for the identification of studies

#### 2.2.1 Electronics searches

We will search the following Electronic databases from their inceptions to April 2020: PubMed, the Cochrane Library, Embase, the China National Knowledge Infrastructure, Wanfang Database, Chinese Science and Technology Periodical Database, and Chinese Biomedical Literature Database. Search terms consist of disease (cervical vertigo, dizziness, cervical spondylopasis of the vertebroarterial type) and intervention (acupotomy, needle knife, acupotomology, needle scalpel) and research types (randomized controlled trial, random trials, controlled clinical trial). The PubMed search strategy is shown in Table 1

**Table 1:**
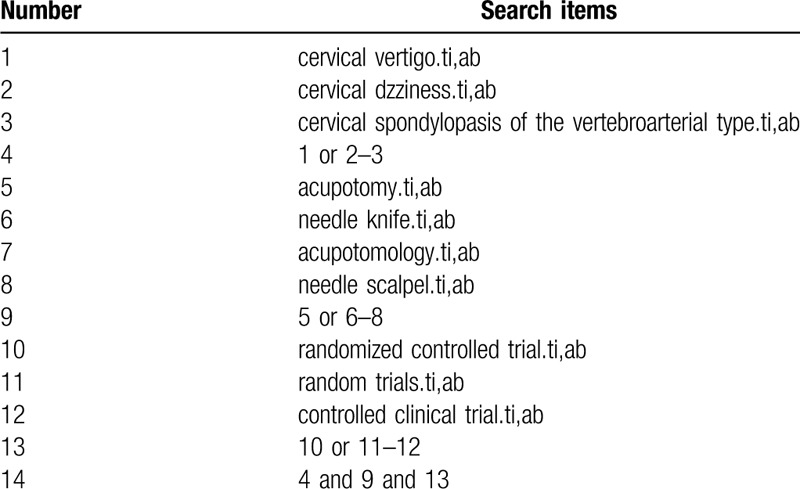
Search strategy used in PubMed database.

#### 2.2.2 Search for other resources

We’ll also check the reference lists of eligible articles obtained from additional studies. In addition, the international clinical trials registry platform, dissertation, and gray literature will also be searched to identify systematic reviews related to acupotomy for CV. The relevant conference papers, journals will be retrieved manually.

### 2.3 Data collection and analysis

#### 2.3.1 Selection of studies

**Figure 1:**
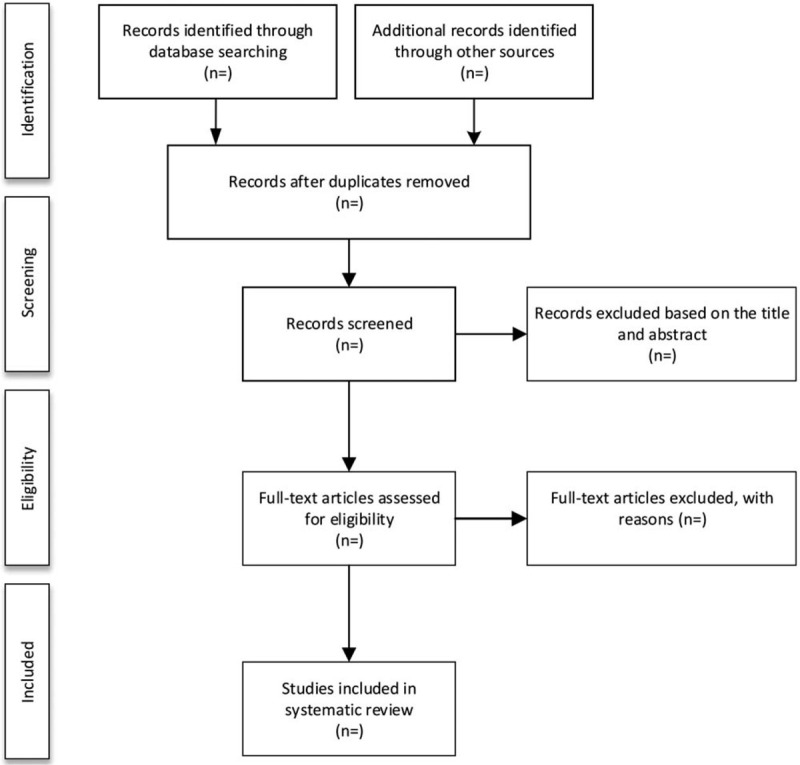
Flow diagram of study selection process.

Researchers will import the literature retrieved to the EndNote X7 and eliminate the duplicate data. After that, 2 reviewers will assess the summaries and titles independently. Irrelevant citations will be excluded. If they could not determine whether to incorporate the study, the full texts of the articles will be obtained. Two reviewers will independently assess the eligibility of these articles against the inclusion and exclusion criteria. Issues will be resolved by agreement after discussion with a third reviewer. The study selection procedure is shown in Figure 1.

#### 2.3.2 Data extraction and management

Two reviewers will independently extract relevant data from the eligible studies, and any disagreement will be settled through discussion with a third reviewer. The extracted study data will mainly included the first author, year of publication, study location, baseline characteristics for participants, sample size, intervention, duration of intervention, follow-up, outcome measurement indexes, and adverse events. If necessary, we will contact the trial author for further information.

### 2.4 Risk of bias assessment

Two reviewers will independently evaluate the risk of bias (ROB) in each included literature according to the Cochrane risk of bias assessment tool.^[16]^ The contents will include:

1. random sequence generation;
2. allocation concealment;
3. blinding of participants and personnel;
4. blinding of outcome assessment;
5. incomplete outcome data;
6. selective reporting; and
7. other sources of bias.

For each item, ROB was graded as high, low, or unclear. If the evaluation results were inconsistent, issues were resolved by rechecking the source papers and further discussions with the third reviewer.

### 2.5 Quantitative data synthesis and statistical methods

#### 2.5.1 Quantitative data synthesis

RevMan 5.3 software will be applied in this meta-analysis. For continuous variables, when outcomes are measured by the same scale, results will be reported as mean differences (MDs) with 95% confidence intervals (CIs); when outcomes are measured by different scales, results will be reported as standardized mean differences (SMDs) with 95%CI. Categorical data will be calculated with the risk ratio (RR) and 95%CI.

#### 2.5.2 Assessment of heterogeneity

Heterogeneity will be evaluated by Chi-Squared test and Higgins *I*^*2*^ test; when *I*^2^ ≤ 50% and *P* > .10, the study will not be considered heterogeneous and the fixed effect model will be used; otherwise, the random effect model will be applied.

#### 2.5.3 Assessment of reporting biases

Publication bias will be estimated by funnel plot analysis if sufficient studies are included (more than 10 studies). When the image is not clear, the Stata14.0 statistical software will be quantified using the Egger test.

#### 2.5.4 Subgroup analysis

If a substantial heterogeneity is detected in the included trials, we will conduct a subgroup analysis based on different control groups.

#### 2.5.5 Sensitivity analysis

If possible, a sensitivity analysis will be performed to verify the robustness of the review conclusions. The impact of methodological quality, sample size, and missing data will be assessed. In addition, the analysis will be repeated after the exclusion of low methodological quality studies.

#### 2.5.6 Grading the quality of evidence

The quality of evidence will be assessed based on the grading of recommendations assessment, development, and evaluation system, includes 4 levels: high, moderate, low, or very low.^[17,18]^

## 3 Discussion

Acupotomy is a new minimally invasive treatment method combining acupuncture and scalpel. It uses acupuncture theory as the guiding ideology, not only can achieve the stimulation effect of acupuncture, but also play the role of cutting and peeling of scalpel.^[12,14]^ Therefore, it is widely used in clinical treatment. Studies have shown that acupotomy can effectively reduce the symptoms of CV,^[15]^ but its efficacy has not been evaluated scientifically and systematically. The purpose of this study is to evaluate the clinical efficacy and safety of acupotomy treatment for CV patients. The conclusions drawn by this study may provide evidence-based medical advice for the treatment of CV with acupotomy. However, this study may also have some potential limitations. First, there may be a risk of heterogeneity in the selection of acupoints, insertion depth, and operation frequency during acupotomy treatment. Second, the reliability of the results largely depends on the comprehensiveness and methodological quality of the main studies included in this review.

## Author contributions

**Data curation:** Rongfang Xie, Jianyu You.

**Formal analysis:** Rongfang Xie, Jianyu You.

**Investigation:** Rongfang Xie, Jianyu You.

**Methodology:** Rongfang Xie.

**Project administration:** Jianyu You,Liting Liu.

**Software:** Liting Liu.

**Supervision:** Chunhua Huang.

**Validation:** Chunhua Huang, Yu Liang.

**Visualization:** Liting Liu, Yu Liang.

**Writing – original draft:** Rongfang Xie, Jianyu You.

**Writing – review & editing:** Chunhua Huang.
